# Resveratrol suppresses body mass gain in a seasonal non-human primate model of obesity

**DOI:** 10.1186/1472-6793-10-11

**Published:** 2010-06-22

**Authors:** Alexandre Dal-Pan, Stéphane Blanc, Fabienne Aujard

**Affiliations:** 1 Mécanismes Adaptatifs et Evolution, UMR 7179 Centre National de la Recherche Scientifique, Muséum National d'Histoire Naturelle, Paris, France; 2 Institut Pluridisciplinaire Hubert Curien, UMR 7178 CNRS Université de Strasbourg, Strasbourg, France

## Abstract

**Background:**

Resveratrol, a natural polyphenolic compound, was shown to protect rodents against high-fat-diet induced diabesity by boosting energy metabolism. To the best of our knowledge, no data is yet available on the effects of resveratrol in non-human primates. Six non-human heterotherm primates (grey mouse lemurs, *Microcebus murinus*) were studied during four weeks of dietary supplementation with resveratrol (200 mg/kg/day) during their winter body-mass gain period. Body mass, spontaneous energy intake, resting metabolic rate, spontaneous locomotor activity and daily variations in body temperature were measured. In addition, the plasma levels of several gut hormones involved in satiety control were evaluated.

**Results:**

Resveratrol reduced the seasonal body-mass gain by concomitantly decreasing energy intake by 13% and increasing resting metabolic rate by 29%. Resveratrol supplementation inhibited the depth of daily torpor, an important energy-saving process in this primate. The daily amount of locomotor activity remained unchanged. Except for an increase in the glucose-dependent insulinotropic polypeptide, a gut hormone known to promote mobilization of fat stores, no major change in satiety hormone plasma levels was observed under resveratrol supplementation.

**Conclusions:**

These results suggest that in a non-human primate, resveratrol reduces body-mass gain by increasing satiety and resting metabolic rate, and by inhibiting torpor expression. The measured anorectic gut hormones did not seem to play a major role in these observations.

## Background

Obesity stems from a prolonged imbalance between the level of energy intake and energy expenditure, with the resultant surplus being stored as lipids predominantly in adipose tissue, but also in muscle and liver tissue, triggering features of the metabolic syndrome. Understanding the factors which regulate both energy intake and expenditure, such as environmental/lifestyle manipulations or pharmaceuticals, is an important step towards developing obesity treatments.

The physiological benefits of resveratrol (3,4',5-trihydroxystilbene or RSV), a natural polyphenol, are currently under intensive investigation. Resveratrol is produced by plants in response to infection by the pathogen *Botrytis cinerea *[1]. It is also induced in response to a variety of stress conditions (climate, exposure to ozone, sunlight and heavy metals) [2]. Presently, it has been detected in more than 70 plant species, including grapes, peanuts, berries and pines [2]. Resveratrol is able to activate nicotinamide adenosine dinucleotide-dependent deacetylase SIRT1 (Silent mating type Information Regulation 2 homologue Type 1), one of the seven mammalian sirtuins [3,4] involved in glucose homeostasis and lipid metabolism. This activation of SIRT1 by RSV seems to prevent obesity by inducing oxidative mitochondrial metabolism [5] and reduced insulin resistance [6] in mice on a high-caloric diet. In the same way, RSV minimizes hyperglycaemia in diet-induced obese and diabetic mice [7], and dyslipidemia in the experimental model of obese Zucker rats. These rodent models are widely used as animal models of obesity and type 2 diabetes which present many features of the human metabolic syndrome [8]. The validity of information obtained in primates, particularly in humans, is still under debate because the experimental protocols used in the preliminary studies were quite different from each other [9]. Moreover, recent works have demonstrated that RSV supplementation mimics caloric restriction in mice [10], suggesting that RSV could be a good candidate for the development of obesity therapies.

However, most RSV studies focused on changes in energy expenditure and its related cellular mechanisms [11,12] and were mainly obtained from genetically modified mouse models of obesity [13] or in the context of a high-fat diet [14,11]. The effects of RSV on spontaneous food intake and its regulation are still unclear and still unknown in primates.

The aim of the present study was thus to determine the effects of a four-week RSV supplementation on energy metabolism and spontaneous food intake in a non-human primate, the grey mouse lemur (*Microcebus murinus*), which demonstrates seasonal spontaneous obesity. Grey mouse lemurs present high photoperiod-dependent variations in body mass associated with energy saving mechanisms, in particular a phase of heterothermia during their daily resting period. When exposed to a short photoperiod, mouse lemurs display a fast and linear body-mass gain (about 6-8 g/week) associated with a high caloric intake. In just a few weeks, a near 100% increase in body mass is observed due to high levels of fat storage, while the duration and depth of daily hypothermia bouts increase [15,16].

The effect of RSV supplementation on body mass, resting metabolic rate (RMR), daily body temperature (Tb) variations and spontaneous food intake in the grey mouse lemur was explored to investigate of the hypothesis that RSV supplementation will affect the time courses of pre-wintering fattening mechanisms in this primate. Because previous studies on mouse lemurs have suggested that several gut hormones may be related to the pre-wintering fattening phase and to the daily heterothermia expression [17], we also tested the effects of RSV supplementation on several gut-derived peptide hormones that are involved in numerous aspects of fuel homeostasis [18]. We measured glucagon-like peptide 1 (GLP-1), pancreatic polypeptide (PP), peptide YY (PYY) and glucose-dependent insulinotropic polypeptide (GIP). Glucagon-like peptide 1 and PYY were shown to decrease energy expenditure and food intake [19]. Conversely, PP and GIP induced an increase in energy expenditure, motor activity and mobilization of fat stores [20]. Consequently, possible relationships between the key parameters involved in energy balance regulation and gut hormones in grey mouse lemurs were also investigated.

## Results

**Figure 1:**
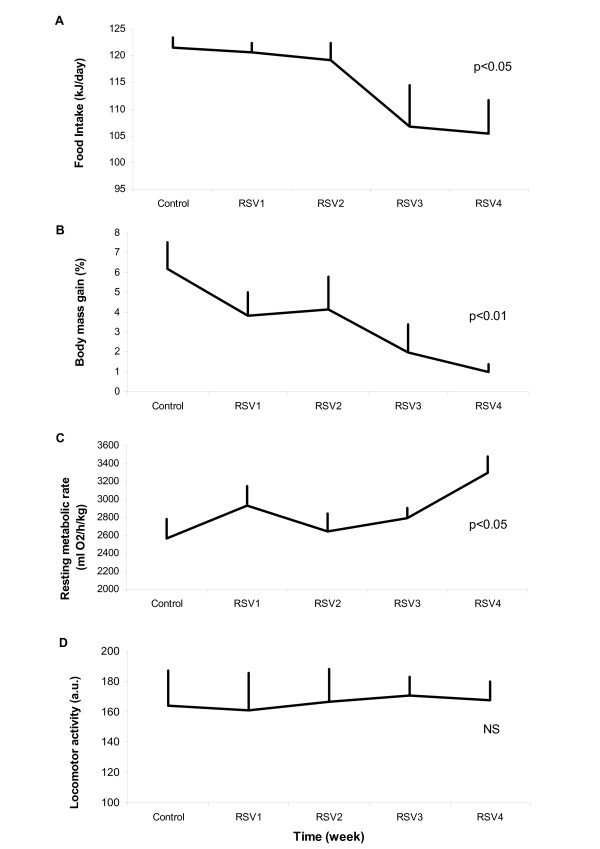
**Resveratrol effects on parameters of energy balance**. Six male mouse lemurs were observed during one week of *ad libitum *feeding (Control), followed by four weeks of RSV supplementation to the *ad libitum *feeding (RSV1, 2, 3 and 4). The curves represent the evolution with time of food intake (A), body-mass gain (B), resting metabolic rate (RMR) (C) and daily amount of locomotor activity (LA) (D). Values are given as mean ± SEM. Resveratrol supplementation induced a significant decrease in food intake and body-mass gain associated with an increase in RMR. The amount of LA was not modified by the treatment.

**Figure 2:**
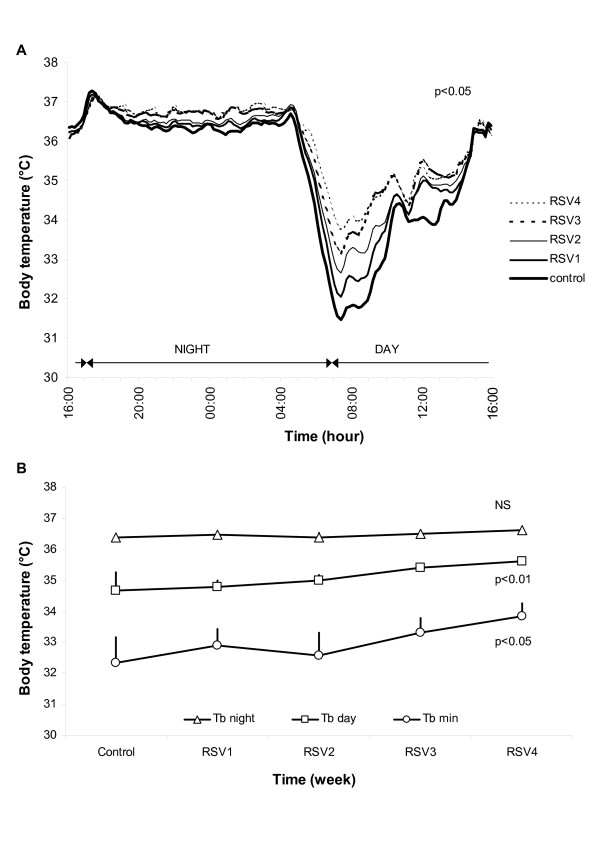
**Resveratrol effects on body temperature of the animals**. Six male mouse lemurs were observed during one week of *ad libitum *feeding (Control), followed by four weeks of RSV supplementation to the *ad libitum *feeding (RSV1, 2, 3 and 4). Panel (A) represents the average daily profiles of the Tb of one representative mouse lemur during the control week and during each week of RSV supplementation. Panel (B) illustrates the evolution of the diurnal (Tb day), nocturnal (Tb night) and minimal (Tb min) body temperatures according to the duration of treatment. Values are given as mean ± SEM. Resveratrol supplementation induced a clear reduction of the phase of daily torpor revealed by the significant increase in Tb day and Tb min values over the course of treatment.

During the control week prior to RSV supplementation, the studied animals presented an average body-mass gain of 6.2 ± 1.3% (1.2 g/d) from their initial mass (133 ± 4 g). At the end of the four weeks of RSV supplementation, the body-mass gain was gradually suppressed to 1.0 ± 0.3% (0.5 g/d, F = 14.0, df = 4, p = 0.007, Figure 1A). A 13% decrease in spontaneous food intake was noted from the third week of RSV treatment (from 121.4 kJ/d to 104.7 kJ/d, F = 10.4, df = 4, p = 0.035, Figure 1B). The RMR increased by 29% after four weeks of RSV treatment compared to the control period (F = 12.7, df = 4, p = 0.013, Figure 1C). Total daily locomotor activity (LA) was not modified by RSV treatment (164 ± 23 a.u. in the control week vs. 168 ± 12 a.u. in the fourth week of treatment, F = 0.4, df = 4, p = 0.980, Figure 1D). A representative profile of the weekly average of variations in Tb of one mouse lemur is represented in Figure 2A. The mouse lemurs exhibited a significant increase in their mean Tb during the diurnal phase (Tb day) with a gain of 1.0 ± 0.3°C between the control and the fourth week of treatment (34.7 ± 0.5°C vs. 35.6 ± 0.1°C, F = 14.3, df = 4, p = 0.006). The minimal Tb value (Tb min) of the animals increased by 1.5 ± 0.6°C between the control and the fourth week of treatment (32.4 ± 0.8°C vs. 33.9 ± 0.4°C, F = 11.1, df = 4, p = 0.026). The amplitude of the phase of daily hypothermia was greatly reduced in response to RSV supplementation. On the other hand, no significant change in the mean Tb during the active nocturnal phase (Tb night) was found in response to RSV supplementation (36.4 ± 0.1°C vs. 36.6 ± 0.1°C, F = 3.3, df = 4, p = 0.504) (Figure 2B).

No significant time-course change was reported for GLP-1, PP and PYY levels. Only the GIP level was significantly increased by RSV after four weeks of supplementation (104 ± 14 pg/ml in the control week vs. 175 ± 39 pg/ml in the fourth week, F = 11.6, df = 4, p = 0.021) (Table 1). Although trends are visible, the increased inter-individual variability observed during the treatment (from 21% to 61%, F = 14.3, df = 4, p = 0.002) did not allow any significant conclusion to be reached. Lastly, no significant correlation could be found between the variations in gut hormones and the variations in body-mass gain, food intake or RMR (Table 2).

**Table 1:** Resveratrol effects on the levels of the different gut hormones tested during the four weeks of treatment.

Gut hormones	Control	RSV1	RSV2	RSV3	RSV4	Friedman test
**GIP**	104 ± 14	123 ± 25	168 ± 28	187 ± 33	175 ± 39	F = 11.6, df = 4, p = 0.021
**GLP-1**	673 ± 25	705 ± 34	899 ± 139	1088 ± 293	1080 ± 295	F = 0.7, df = 4, p = 0.949
**PP**	205 ± 17	206 ± 22	193 ± 18	148 ± 36	140 ± 33	F = 4.2, df = 4, p = 0.385
**PYY**	298 ± 11	299 ± 16	329 ± 23	293 ± 47	261 ± 57	F = 1.8, df = 4, p = 0.779

The gut hormone levels investigated were glucose-dependent insulinotropic polypeptide (GIP), glucagon-like peptide-1 (GLP-1), pancreatic polypeptide (PP) and peptide YY (PYY). RSV1, 2, 3 and 4 represent the four successive weeks of RSV treatment. Values are expressed in pg/ml and they are given as mean ± SEM (n = 6)

**Table 2:** Correlations between the variations in gut hormone levels and the variations in energy parameters.

Gut hormones	Body mass gain	Food intake	Resting metabolic rate
**GIP**	R = 0.260, p = 0.658	R = 0.260, p = 0.658	R = -0.200, p = 0.714
**GLP-1**	R = 0.086, p = 0.919	R = 0.543, p = 0.297	R = 0.143, p = 0.803
**PP**	R = -0.314, p = 0.564	R = 0.657, p = 0.175	R = 0.486, p = 0.356
**PYY**	R = -0.029, p = 1.000	R = 0.029, p = 1.000	R = 0.086, p = 0.919

Correlations were analysed between gut hormone levels (glucose-dependent insulinotropic polypeptide (GIP), glucagon-like peptide-1 (GLP-1), pancreatic polypeptide (PP) and peptide YY (PYY)) and variations in body mass gain, food intake and resting metabolic rate between the control week and the fourth week of RSV supplementation (all results are non significant) (n = 6).

## Discussion

The main findings of this study were: 1) a four-week resveratrol supplementation had significant effects on energy metabolism in the mouse lemur, characterized by a reduction in seasonal body-mass gain associated with an increase in resting metabolic rate and a decrease in food intake; 2) the response to resveratrol supplementation mainly involved a strong reduction of daily heterothermia expression with no change in the daily amount of locomotor activity; 3) despite the fact that glucose-dependent insulinotropic polypeptide (GIP) values increased during resveratrol supplementation, no correlation was evidenced between the other tested gut hormones and changes in energy balance parameters.

Resveratrol supplementation was given to mouse lemurs at the very time of seasonal fattening induced by exposure to short day lengths. This period corresponds to an increase in both spontaneous food intake and frequency of deep torpor, leading to fast body mass gain through fat accumulation. Moreover, the resting metabolic rate and locomotor activity decrease, reinforcing the energy saving processes. Resveratrol supplementation appeared to limit all these energy saving mechanisms by acting more specifically on food intake and daily hypothermia bouts without change in locomotor activity.

The observed decrease in food intake in mouse lemurs cannot be related to the palatability of resveratrol as the mouse lemurs were not found to be averse to resveratrol prior to the experiment. It is more likely that an aversive palatability of resveratrol would have led to an immediate reduction in food intake which was not observed. However, the effects on food intake might have been related to the dose of resveratrol given. The dosage level administered in this study (200 mg/kg) was selected from studies in the literature on rodents, and was intermediate between the 40 mg/kg of Baur et al. [14] and the 400 mg/kg of Lagouge et al. [12]. This dose was carefully mixed in the food and, since the animals tended to eat less than provided, the actual ingested dose of resveratrol was below 200 mg/kg. The amount of resveratrol ingested by the animals was 96.1% in the first week and 84% in the fourth week (corresponding to an equivalent of 192 mg/kg/day and 168 mg/kg/day, respectively). These amounts, based on the existing literature, appeared sufficiently high to be effective in mammals [21-23]. However, the observed satiety in resveratrol-supplemented mouse lemurs may be a sensibility response specific to this species or to primates.

Although the change in food intake could explain the reduction of seasonal body-mass gain, resveratrol supplementation significantly affected the body temperature patterns in the mouse lemur. Average values of energy savings by daily torpor in this species have been estimated to be about 40-70% as compared to the maintenance of normothermia [24,25]. Under resveratrol supplementation, the depth and the duration of daily torpor decreased and were associated with an increase in both body temperature and resting metabolic rate during the daily resting period. In contrast, body temperature values during the night active phase were not modified, and nor was locomotor activity. The decrease in energy savings during the daily diurnal rest would thus contribute to the reduction in body-mass gain under resveratrol supplementation.

No such effect of resveratrol has been shown in mice, for which no change in food intake or body temperature was observed [11,13]. However, these data originate from experimentally-induced obesity and it is likely that the responses observed in mouse lemurs rely on specific life-history traits of this primate, which has developed high seasonal energy saving mechanisms and exhibits pre-wintering spontaneous obesity.

Resveratrol activates mammalian sirtuins, more specifically SIRT1, which is involved in glucose homeostasis and lipid metabolism. In mice exposed to a high-fat diet or in ob/ob mice, resveratrol was shown to protect mice from diet-induced metabolic disorders [12,13,26] by mimicking some effects of caloric restriction. Likewise, resveratrol and assimilated SIRT1 activators have been proposed as potential treatments for type 2 diabetes in mice models [14,12,27,28]. The effects of resveratrol have been suggested to depend on an increase in oxidative mitochondrial metabolism [14] and on enhanced fat oxidation [11], leading to an increase in energy expenditure. Interestingly, SRT1720, which activates SIRT1 by four fold in comparison to resveratrol, induces a significant decrease in fat mass without a change in food intake in mice [11]. In the mouse lemur, concomitantly to an increase in energy expenditure through decreasing hypothermia bouts and increasing metabolism, resveratrol might have a direct negative effect on fat storage.

Due to their involvement in satiety and satiation processes [18,29,30], several gut hormones have been measured in resveratrol-supplemented mouse lemurs. No significant change was recorded after four weeks for the glucagon-like peptide 1 and the peptide YY, two gut hormones involved in the reduction of food intake and energy expenditure [31-34]. Conversely, the two other gut hormones are known to induce an increase in energy expenditure. The pancreatic polypeptide increases satiety, thus leading to a loss in body mass [20] and the glucose-dependent insulinotropic polypeptide (GIP) was shown to induce an increase in exploratory behaviour and performance in some motor function tests in a transgenic mouse that over-expressed GIP [35]. Only GIP significantly increased with resveratrol supplementation. Data emerging from studies in animal models and cultured human fat cells support a physiological role for GIP in fat cell metabolism, leading to the mobilization of fat stores [17,36]. Such effects might have contributed to the reduction in body-mass gain by limiting fat storage in supplemented mouse lemurs. The observed effect of resveratrol on food intake in the mouse lemur appears to be independent of changes in anorexic gut hormones, but the high observed inter-individual variability in our study does not allow definite conclusions to be reached. Moreover, because sampling was performed at a single point in fasted animals during the diurnal rest, changes may have been missed.

Resveratrol has been studied as a potential mimetic of caloric restriction [4]. However, caloric restriction decreases the metabolic rate [37], whereas resveratrol had the opposite effect [12]. From previous studies using moderate caloric restriction in wintering mouse lemurs [17], the effects of resveratrol significantly differed from changes observed in caloric-restricted animals. In the mouse lemurs, caloric restriction led to an increase in the duration and depth of daily torpor bouts. These body temperature adjustments were efficient at preventing body-mass loss and were not associated with changes in gut hormones [17].

## Conclusion

In conclusion, we demonstrated for the first time the short-term effects of resveratrol on the metabolism of an heterothermic primate. Resveratrol activates energy expenditure by inducing an increase in resting metabolic rate and a decrease in torpor patterns that play key roles in energy saving in this primate. Moreover, resveratrol had a satiety effect in this primate that reduced their spontaneous food intake. All of these changes participated in the limitation of pre-wintering fattening processes in which the increase in the glucose-dependent insulinotropic polypeptide levels would play an additive role. These results provide novel information on the potential effects of resveratrol on energy metabolism and control of body mass in a primate.

## Methods

### Animals and animal care

We used six male grey mouse lemurs (*Microcebus murinus*, Cheirogaleidae, primates) born in a laboratory breeding colony in Brunoy, France (Agreement # 962773) from a population originally caught 40 years ago on the southwest coast of Madagascar. The animals were 3-year-old adults and presented a mean ± standard error of the mean (SEM) body mass of 133 ± 4 g. Conditions were constant in respect to ambient temperature (25°C), relative humidity (55%), and *ad libitum *food availability. Behavioural and physiological seasonal changes in mouse lemurs are dependent on the photoperiod and are reproduced in captivity using an artificial photoperiodic regimen. In the breeding colony, animals were exposed to an artificial photoperiodic regimen consisting of alternating six-month periods of Malagasy winter-like short-day lengths (light:dark 10:14) and Malagasy summer-like long-day lengths (light:dark 14:10). Animals were studied during the five weeks following the shift from long to short day lengths corresponding to the pre-wintering fattening period. To minimize social influences, the animals were housed individually in cages (0.4 × 0.4 × 0.6 m) provided with nesting materials, and were separated from each other by wooden partitions. Each week and just before the daily food allotment, animals were weighed inside their nest box, to avoid any stress, using a balance accurate to 1 g.

**Figure 3:**
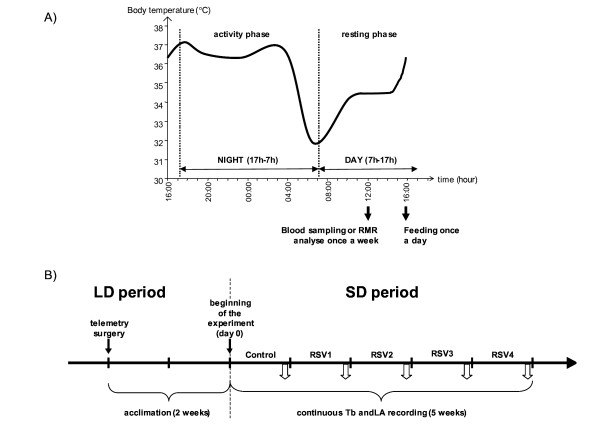
**Experimental timetable**. A) The activity phase (17h-7h) and the resting phase (7h-17h) are represented in this schematic profile of body temperature variations of one animal during one day. The animals were followed for five weeks: one control week and four weeks with RSV supplementation (200 mg/kg/day). The mouse lemurs were fed daily one hour before the beginning of their active phase during the five-week study. Blood sampling and measurement of the resting metabolic rate (RMR) were carried out once a week (each one on a different day) during the control period and each week of RSV treatment during their daily resting period, 4-6 h after the beginning of the light period to minimize the influence of circadian variations. B) This long-term experimental time line shows when the telemetry surgery, acclimation and first experiments were performed. The telemetry surgery was performed two weeks before the beginning of the short day period to allow animals to recover before beginning the experiment. The white arrows represent the moment when blood sampling, measurement of resting metabolic rate (RMR) and body weight were undertaken (once a week). The body temperature (Tb) and locomotor activity (LA) were continuously recorded during the five weeks from the shift to short day period.

All animals were fed *ad libitum *during one week, with fresh fruit (bananas and apples) and a mixture of cereals, milk and eggs, providing them with a total of 126 kJ per day. The cereals were composed of 60% carbohydrates, 10% proteins and 30% lipids. The cereals were primarily wheat flour (96%). The mixture was introduced 1 h before the beginning of the animal's nocturnal active phase (Figure 3A). Water was always given *ad libitum*. During the following four weeks (RSV1, RSV2, RSV3 and RSV4), the animals were fed the same as above but 200 mg/kg of RSV (Sequoia Research Products, United Kingdom) per day was added to the mixture. Prior to the study, we monitored the food intake of the six animals presented with a choice between a bowl of control food and a bowl of food supplemented with 200 mg/kg/day of RSV. Food was provided *ad libitum *in both bowls. Food intake was assessed by weighing the daily leftovers and correcting for water evaporation (calculated previously with the control mixture under the same environmental conditions). The average daily amounts of food eaten were similar between control and RSV supplemented groups (t = 1.738, df = 5, p = 0.143), evidencing no significant difference in the palatability of the mixtures. To determine the exact quantity of food ingested by the animals, daily leftovers were weighed and corrected for water evaporation as with the palatability control. All the procedures were carried out in accordance with the European Communities Council Directive (86/609/EEC). All the experiments were done under personal licence to experiment on mouse lemurs, delivered by the Ministry of Education and Science after approval of a local Ethics Committee (n° 91-305, issued 20 July, 2006).

### Resting metabolic rate

Oxygen consumption was measured in a closed indirect calorimetry system. Measurements on post-absorptive animals were made daily during their resting period, 4-6 h after the beginning of the light period to minimize the influence of circadian variations (Figure 3A). The animals were trained to nest in 2.5 l opaque respiratory chambers with a woven floor to absorb any urine. The respiratory chamber was placed in a cabinet at a controlled ambient temperature of 25.0 ± 0.5°C, a value within the thermoneutral zone defined for the mouse lemur [38]. After a 20 min habituation phase with a constant air flow (2 l/min) drawn through the respirometry chamber from bottom to top, the chamber was closed for a 40-min period. The air was dried prior to analysis using a silica gel. The VO_2 _consumed by the animal was calculated from initial and final concentrations of O_2 _in the chamber which were measured from dried gas using a SERVOMEX 570A paramagnetic gas analyser (Servomex Ltd., England) (accuracy 0.01% O_2_). Calculations were made in Standard Temperature and Pressure, Dry and, the daily atmospheric humidity was accounted for. The oxygen analyser was routinely calibrated with N_2 _and atmospheric air assuming 21.00% O_2 _as recommended by the manufacturer. Oxygen consumption was expressed as ml O_2_/h. The animals were weighed before each measurement. Oxygen consumption was corrected for the body weight of the animal [39]. Body temperature was not measured before the experiment to avoid disturbing sleeping animals but males never enter deep torpor at an ambient temperature of 25°C [40]. However, O_2 _consumption at 25°C cannot be defined as basal because the body temperature varies in the grey mouse lemur, thus values were referred to as resting metabolic rate (RMR) [15]. The values are given as mean ± SEM.

### Recording of locomotor activity and body temperature

The grey mouse lemur is a nocturnal species exhibiting high levels of locomotor activity (LA) during the dark period and complete rest during the light period. Under exposure to short days, the daily rhythm of body temperature (Tb) in the mouse lemur is characterized by high Tb values during the active period and, before the onset of the light phase, a rapid and linear drop in Tb reaching minimal values after 3-4 hours. This hypothermia bout is followed by a spontaneous re-warming to normothermic Tb levels until the following dark phase (Figure 3A).

The recording of LA and Tb was obtained by telemetry at a constant ambient temperature of 25°C. A small telemetric transmitter weighing 2.5 g (model TA10TA-F20, DataScience Co. Ltd, Minnesota, USA) was implanted into the visceral cavity under ketamine anaesthesia (Imalgene, 100 mg/kg ip, Merial, France). Telemetry surgeries were performed 15 days before the beginning of the short day period. After surgery, the animals were returned to their home cage and allowed to recover for 15 days before starting the experiment and the continuous recording of Tb from the beginning of the short day period. Total recovery was checked by visual inspection to ensure complete healing of the surgical incision and by verification of a stable daily pattern of Tb variations. Recording began in the first week following the shift to the short photoperiod (Figure 3B). A receiver board was positioned in the cage and LA (expressed in arbitrary units, a.u.) was continuously recorded during this interval by two antennas located in the receiver board which detected vertical and horizontal movements (X-Y coordinate system, Dataquest Lab Pro v. 3.0, Data Science Co. Ltd, Minnesota, USA). The Tb (in°C) of the animals was recorded every 10 min. The following parameters were analysed: daily amount of LA, mean Tb during the active nocturnal phase (Tb night), mean Tb during the resting diurnal phase (Tb day) and the minimal Tb value (Tb min).

### Hormonal assays

Blood sampling was carried out once a week during the control period and in each week of RSV treatment 4-6 h after the beginning of the light period to minimize the influence of circadian variations (Figure 3A). The blood samples were taken via the saphenous vein, without anaesthesia, before food allotment. The volume of blood sampled was 100 μl and that represented less than 2% of the total blood volume of the animals. The blood samples were centrifuged at 7000 rpm at 4°C for 30 min. The plasma was stored at -20°C according to the assay procedure. Levels of glucagon-like peptide 1 (GPL-1), pancreatic polypeptide (PP), peptide YY (PYY) and glucose-dependent insulinotropic polypeptide (GIP) were measured in duplicate using the human gut hormones multiplex panel (LincoplexTM Multiplex Assays, Bioscience) and Luminex technology at the Saint Antoine Hospital in Paris dedicated to micro-assays in small animals (IRSSA, INSERM, IFR65, France), as previously described [17]. The average recoveries of known quantities of standard peptides (taken at the low, medium and high concentrations given in the assay) added to mouse lemur plasma were 89% for GIP, 83% for GLP1, 88% for PP and 107% for PYY. Inter-assay and intra-assay coefficients of variation were < 19% and < 11%, respectively.

### Statistics

All values are expressed as mean ± SEM. We used the Friedman test to assess significant variations in all of the studied parameters. Comparisons were considered to differ significantly when p < 0.05. Possible correlations between the energy balance parameters and gut hormone levels were assessed by Spearman's correlation test. All statistical computations were performed using SYSTAT for Windows (V9, SPSS Inc., Illinois, USA).

## Abbreviations

a.u.: arbitrary unit; GIP: glucose-dependent insulinotropic polypeptide; GLP-1: glucagon-like peptide 1; LA: locomotor activity; PP: pancreatic polypeptide; PYY: peptide YY; RMR: resting metabolic rate; RSV: resveratrol; RSV1: first week of RSV supplementation; RSV2: second week of RSV supplementation; RSV3: third week of RSV supplementation; RSV4: fourth week of RSV supplementation; SEM: standard error of the mean; SIRT1: Silent mating type Information Regulation 2 homologue Type 1; Tb: body temperature; Tb night: mean Tb during the active nocturnal phase; Tb day: mean Tb during the resting diurnal phase; Tb min: minimal Tb value.

## Authors' contributions

AD performed the experimental protocol, analysed the data and drafted the manuscript. SB participated in the design of the study and drafted the manuscript. FA conceived the study, participated in its design and coordination, performed the statistical analyses and drafted the manuscript. All authors read and approved the final manuscript.
